# Case Report: Not all recurrent “idiopathic” anaphylaxis is idiopathic

**DOI:** 10.3389/falgy.2025.1661992

**Published:** 2025-10-02

**Authors:** Sally Mahgoub Khalil, Sherin Rahim, Hassan Mobayed, Maryam Ali Al-Nesf, Sami Bahna

**Affiliations:** 1 Allergy and Immunology Division, Department of Medicine, Hamad Medical Corporation, Doha, Qatar; 2 Allergy and Immunology Division, Louisiana State University Health Sciences Center, Shreveport, LA, United States

**Keywords:** anaphylaxis, carmine (E 120), food color, food additive, idiopathic anaphylaxis

## Abstract

**Introduction:**

Anaphylaxis is the most severe manifestation of systemic immediate hypersensitivity, yet the underlying trigger often remains elusive. When routine history and allergy testing fail to identify a cause, the condition is classified as idiopathic anaphylaxis. Food additives, although uncommon culprits, may be overlooked, particularly in atopic individuals.

**Methods:**

We report a case of a 39-year-old woman with recurrent anaphylaxis initially diagnosed as idiopathic. Standard allergy testing, including extended skin prick and specific IgE panels, was negative. Due to a temporal association with restaurant-prepared food, an additive hypersensitivity was suspected. A detailed dietary history and targeted skin prick testing were employed using both commercial and in-house preparations of food colorants.

**Results:**

SPT was positive for carmine-containing red food colorants, including a commercially available gel and a prepared cochineal extract. Control subjects tested negative. sIgE to carmine was equivocal. The patient was educated about allergen avoidance and has remained symptom-free following elimination of carmine from her diet, cosmetics, and medications.

**Conclusion:**

This case underscores the importance of considering food additives, particularly carmine, in patients with unexplained anaphylaxis. Structured re-evaluation, patient-guided dietary review, and custom allergen testing may be essential in identifying hidden allergens. Clinicians should be vigilant about uncommon triggers when routine investigations fail to identify the cause.

## 1 Introduction

Anaphylaxis is the most severe form of systemic immediate hypersensitivity, requires prompt aggressive treatment and dictates setting a prevention plan. Identifying the causative trigger is essential but not always straightforward, particularly through history-taking and/or routine allergy testing for the common causes. Cases are often subsequently labeled as “idiopathic anaphylaxis,” leaving patients at risk of recurrence.

Hypersensitivity to food additives is uncommon in the general population (0.01%–0.23%) but more prevalent in atopic individuals (2%–7%) (1). These reactions can be immunologic, that can be IgE-mediated, cell-mediated or combined, or non-immunologic, corresponding to food intolerances driven by metabolic, pharmacological, toxic, and other unspecified processes (2).

Cochineal dye is a natural colorant derived from dried female cochineal insects (Dactylopius coccus), found on cacti in Central and South America, specifically Peru. The main component of this red dye is carminic acid (CA), which has a molecular weight of 492 Daltons (3, 4). Carmine, a substance derived from CA through hydration and chelation with aluminum or calcium, is employed as an insoluble red dye. Carmine, along with cochineal dye, has global applications as a colorant in food, beverages, cosmetics, and pharmaceuticals (4). Proteins derived from insects, such as those in cochineal dye, can potentially cause IgE-mediated allergic responses (5, 6).

We present the case of an adult with recurrent allergic reactions, including anaphylaxis, in whom the trigger could not be identified by routine allergy evaluation. Guiding the patient in keeping a detailed record of events that preceded every recurrence could suspect food additives and a special allergy revaluation could identify the definitive culprit that could be avoided, and the reaction did not recur. This report, the first from Qatar, emphasizes the need to identify hidden food additives as overlooked causes of severe allergic reactions frequently labeled as idiopathic, with its novelty lying primarily in the clinical insight and regional context.

## 2 Case description

A 39-year-old woman with a medical history of high-grade invasive ductal carcinoma of the right breast, associated with a positive BRCA1 gene mutation, underwent bilateral mastectomy in 2017. She also had bariatric surgery in 2016. Additional history included resolved childhood asthma, intermittent mild urticaria, and allergy to black ant stings.

At the age of 30 (2015), she began experiencing 2–4 episodes of anaphylaxis per year, prompting referral to allergy service at the age of 33, where was labeled as idiopathic and was advised to use an epinephrine autoinjector. She was seen again for reevaluation last year due to significant quality of life affection with multiple emergency visits proximal to each other with anaphylaxis episodes. Her anaphylactic reactions typically manifested as urticarial rash and angioedema, often accompanied by one or more of the following symptoms: chest wheezing, lightheadedness, abdominal pain, and vomiting. Initially, no specific trigger could be identified and foods, medications, insect stings, or physical exertion were all excluded. Most episodes occurred within 30 min of eating, frequently in restaurants. Notably, she never experienced allergic reactions to food prepared at home using fresh ingredients. Two episodes occurred in close proximity to her menstrual cycle.

### 2.1 Initial allergy evaluation

Skin prick tests (SPT) (Greer Laboratories, Lenoir, NC) and specific IgE tests (Thermo Fisher Scientific/Phadia, Uppsala, Sweden) for extended panels of food and inhalant allergens were negative. Total IgE level was 178 kU/L (reference: <114 kU/L) and baseline tryptase level was 4.37 μg/L (reference: <11 μg/L). Based on these findings, the initial diagnosis was idiopathic anaphylaxis.

### 2.2 Initial management

She was advised to carry an epinephrine autoinjector for emergency use during acute reactions, with immediate transfer to the emergency department following administration. She was also instructed to monitor the timing of reactions in relation to her menstrual cycle and to keep a detailed record of events and foods consumed preceding each event.

After the patient had experienced multiple acute reactions, suspicion was directed to red-colored foods, especially those consumed at restaurants, as possible triggers. She tolerated foods of other colors, such as green, yellow, and blue without issues. However, she recalled experiencing reactions after eating certain types of hot dogs, mortadella, and spicy chicken strips, whether purchased frozen or served at restaurants. Similar reactions were observed after ingesting some imported packaged foods that lacked clear labeling. She did not report a consistent association with the menstrual cycle.

After carefully reviewing the ingredients in the suspected foods brought by the patient, it became evident that red food coloring was a primary suspect, along with yellow and orange dyes.

### 2.3 Further allergy evaluation

The patient consented to testing with food dyes by SPT but declined intradermal testing or provocation challenges due to concerns about triggering an anaphylactic reaction. She also agreed to medroxyprogesterone but declined estrogen SPT, given her personal history of breast cancer and potential risks associated with hormone exposure.

### 2.4 Testing material

SPT to assess catamenial anaphylaxis using medroxyprogesterone was negative.

The SPT (7) was conducted using various red food coloring brands available in the region (Table 1). The result of the skin test was taken after 15 min. Dr. Oetker® Food Color Gel-Red test yielded a positive result (wheal: 7 mm, flare: 12 mm). To validate the results, the same test was conducted on two healthy controls, both of whom tested negative. This specific food color contains not only lutein (E161b) and carmine (E120) but also glucose syrup, water, sugar, acidity regulators (citric acid, lactic acid, acetic acid, and sodium lactate), gelling agent (carrageenan); and preservative (potassium sorbate). To confirm that the reaction was due to the red colorants and not other ingredients, SPT was performed using Dr. Oetker® Food Color Gel-Yellow (same manufacturer), sharing the same components except for its colorant (Curcumin E 100). The test yielded a negative result, suggesting that colorants (lutein and/or carmine) were indeed the trigger (Table 1).

**Table 1:** Tested food colorants.

Product name	Colorants (E-numbers)	Additional components	Approx. concentration/dilution	SPT result
Dr. Oetker® Food Color Gel-Red (Dr. Oetker, Bielefeld, Germany)	Lutein (E161b), Carmine (E120)	Glucose syrup, water, sugar, citric acid, lactic acid, acetic acid, sodium lactate, carrageenan, potassium sorbate	Not specified by manufacturer	Positive (Wheal: 7 mm*, Flare: 12 mm)
Foster Clark's® Red Food Color (Foster Clark Products Ltd, San Ġwann, Malta)	Azorubine (E122), Brilliant Blue (E110)	Not specified	Not specified by manufacturer	Negative
Delicio® Food Color Red (Delicio, Mumbai, India)	Sunset Yellow FCF (E110), Allura Red (E129)	Not specified	Not specified by manufacturer	Negative
Dr. Oetker® Food Color Gel-Yellow (Dr. Oetker, Bielefeld, Germany)	Curcumin (E100)	Same as Red Gel except colorant	Not specified by manufacturer	Negative
Positive control: Histamine base 6 mg/ml (histamine dihydrochloride: 10 mg/ml, Jubilant HollisterStier LLC, Spokane, WA 99207, U.S. license No.1272)	NA	NA	6 mg/ml	Positive (10 mm wheal and a 20 mm flare)
Negative control: sterile 50% glycerin sterile 50% glycerin glycerinated saline (Greer Laboratories, Lenoir, NC 28645. U.S. License 308)	NA	NA	–	Negative

*: ≥3 mm wheal is considered positive.

Lutein (E161b) and carmine (E120) were tested separately in subsequent SPT. Since no standardized protocol existed, the SPT solutions were developed using information from previous studies or manufacturer guidance. To prepare the lutein solution, we used Herbadiet® Lutein with Zeaxanthin/Marigold Extract Powder (Rohtak Ltd., India), a nutritional supplement containing 20% lutein and 2% zeaxanthin derived from marigold flowers. The solution was prepared according to the manufacturer's guidelines for daily oral consumption by dissolving 100 mg of the powder in 10 ml of distilled water at ambient temperature. There is no established protocol to prepare the cochineal solution; instead, it was prepared using cochineal granules (Jacquard®, Ltd., Lima, Peru) in accordance with the manufacturer's instructions. The granules were initially ground, and a minimal quantity was dissolved in a limited volume of hot water to produce a dilute concentration of 10 mg/ml, so preventing skin irritation. Table 2 summarizes the inhouse test development and results.

**Table 2:** In-house allergen test preparation.

Substance/Test	Preparation/Method	Final concentration	Result
Cochineal (JacquardÂ® granules)	10 mg/ml solution, hot water, prick-to-prick	10 mg/ml	Positive (Wheal: 15 mm*, Flare: 30 mm)
Lutein (HerbadietÂ®)	100 mg in 10 ml distilled water, ambient temperature, prick-to-prick	10 mg/ml (20% lutein, 2% zeaxanthin)	Negative
Histamine (positive control)	6 mg/ml base (10 mg/ml dihydrochloride)	6 mg/ml	Positive (Wheal: 10 mm, Flare: 15 mm)
Saline (negative control)	50% glycerinated saline	–	Negative

*: ≥3 mm wheal is considered positive.

**Figure 1:**
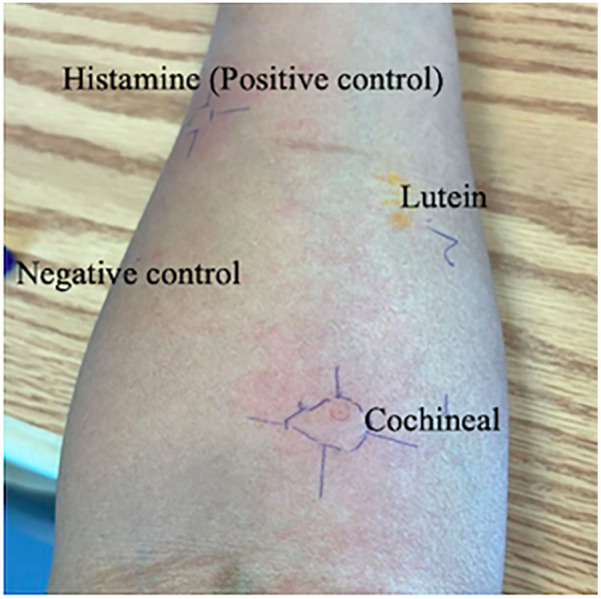
Skin prick test positive to cochineal food colorant but negative to lutein.

Both solutions were entirely dissolved and maintained at room temperature until the time of testing. The SPT was performed using the prick-to-prick method, adhering to the standard protocol (8), and the size of the produced wheal in response to a control or tested substance was measured with positive SPT response defined as 3 mm or greater than negative control. The cochineal solution testing was positive (wheal: 15 mm, flare: 30 mm), while the lutein solution testing was negative (Table 2). SPT for the negative control was negative, and the positive control had a positive result (wheal: 10 mm, flare: 15 mm) (Figure 1). For validation, we performed cochineal SPT for two healthy controls, both of which yielded negative results.

A blood sample was analyzed for IgE specific to Carmine Dye/Red dye Cochineal (Dactylopius coccus) (Red #4) using the ImmunoCAP FEIA method from Mayo Clinic Laboratories, resulting in a value of 0.15 kU/L, which the laboratory classified as equivocal/borderline (0.10–0.34 kU/L). The negative result was defined as <0.10 kU/L, low positive as 0.35–0.69 kU/L, moderate positive as 0.70–3.49 kU/L, and any value exceeding this range as high positive (9).

The patient was advised to strictly avoid exposure to carmine. She was instructed to carefully check labels on all products, including food, cosmetics, and medications, for “cochineal extract” or “carmine” as well as for alternative names of carmine, such as “crimson lake,” “natural red 4,” “C.I. 75470,” and “E120,” which might appear on ingredient lists. A medical alert card was also provided to the patient.

Since her diagnosis, she has been avoiding any food, medication, or cosmetics containing carmine or Cochineal. She avoids items with ambiguous labels. She has been performing quite well, with no additional anaphylactic episodes.

## 3 Discussion

This is the first documented case of food additive-induced anaphylaxis in Qatar. It highlights the diagnostic challenge of identifying hidden allergens, particularly food additives, as culprit triggers in patients with recurrent or idiopathic anaphylaxis (10). The first allergens in cochineal red were identified in 1994, and since then, multiple cases of cochineal-induced anaphylaxis have been reported (11). Carmine protein content is unregulated and may vary significantly between manufacturers, reaching up to 25% (5, 12). In 2009, the FDA mandated that carmine and cochineal extract be explicitly named on the labels of all food and cosmetic products made in the United States (13); however, this requirement does not apply to imported products from countries without mandatory labeling, posing a potential risk.

Food colorants are among the most commonly overlooked additives that have been used since ancient times, with Romans and Egyptians known to color food, beverages, medicines, and other everyday products as early as 1500 B.C.E (14). Today, food colorings continue to be widely used in the production of various items, including baked goods, confectionery, processed meats, dairy products, soft drinks, and more (15). These food coloring can be derived from natural sources, synthetic sources, or synthetic sources equivalent to natural ones (3).

Cochineal dye, also known as carmine, is a natural red colorant derived from the dried bodies of the female Dactylopius coccus insect and among the most well-known food additives that can trigger allergic reactions (11). It has been extensively used since the 16th century in processed food and beverages, pharmaceutical, textile, and cosmetics industries (4, 16). Carmine is now found in products such as sweets, meat products, soft drinks, colored cakes and biscuits, syrups, lipstick and other makeup (13).

Exposure to carmine by ingestion, inhalation, or skin contact can result in allergic reactions that can manifest with a range of symptoms, including redness, hives, angioedema, exacerbation of atopic eczema, difficulty breathing, gastrointestinal symptoms, and anaphylaxis (5). Females exhibit a greater vulnerability to developing an allergic reaction to cochineal color, potentially due to sensitization through cosmetic use (4). Occupational exposure has also been linked to allergic rhinitis, asthma, and hypersensitivity pneumonitis in workers handling natural dyes or spices (17).

Due to the absence of universally standardized diagnostic methods, and labeling inconsistencies across countries, allergic responses to cochineal or carmine are often overlooked, leading to an underestimation of the true prevalence of food color allergies (1).

In our patient, carmine emerged as the likely trigger following extensive evaluation. Initial testing, including SPT and specific IgE to common food and inhalant allergens, was unremarkable. However, a careful clinical history and precise diary documentation of symptoms related to food or other triggers revealed a temporal relationship between her reactions and the consumption of processed foods, particularly those with red coloring. Individuals with atopy are more prone to react adversely to food additives (2), and recognizing food additives as potential triggers is essential, particularly in cases of idiopathic anaphylaxis (18).

SPT for carmine allergen can be done by using dried insects (D. coccus) suspended in tap water at a concentration 10 times their weight (4). Another method involved diluting the powder content of one capsule (0.1 mg/ml NaCl) or a 5% carmine powder with a mixture of saline and glycerol at a ratio of 1:1 to obtain a 1% carmine solution (5, 6) We employed cochineal natural dye granules in our patient's SPT at a concentration of 0.1 mg/ml, which yielded a positive result in our highly sensitive patient. A positive SPT result and a medical history that points to carmine hypersensitivity should be sufficient for establishing a carmine allergy diagnosis. It was advised to measure the sIgE level in the event that a history of allergies was suspected with negative skin test results (4, 5). Our patient's serum specific IgE to carmine was in the equivocal range (0.15 kU/L). The amount of sIgE that might cause allergic reactions can reach 0.19 kU/L (4). However, allergic reactions can happen at much lower concentrations. A Carmine oral challenge was conducted in patients with sIgE levels of 0.01 kU/L; some patients had allergic reactions regardless of SPT results. Therefore, regardless of the carmine SPT result, there is still a chance of an allergic reaction in an individual who suspects a carmine allergy and has s.IgE values between 0.01 and 0.35 kU/L (5). Importantly, specific IgE testing alone is unreliable and should not be used in isolation for diagnosis. The definitive diagnosis can only be achieved through a provocation test with carmine-containing food (2). Other possible diagnoses, such as food-dependent exercise-induced anaphylaxis and hormonal influences, were excluded based on the clinical history, which included the absence of any physical activity following food intake that could induce anaphylaxis. Although hormonal influences were initially considered, further evaluation was not pursued, as most reactions were not consistently related to menstrual timing, and the patient's clear clinical improvement after eliminating carmine reduced the need for additional investigation in this direction. Mast cell activation syndrome was also excluded given the normal baseline tryptase level and the absence of multi-organ involvement outside acute episodes. In this context, food additive hypersensitivity remained the most plausible explanation, supported by reproducible skin test results and sustained symptom resolution after carmine elimination.

Managing carmine or food allergies centers on strict avoidance. Patients must be thoroughly counseled on the wide range of products that may contain carmine, including food, cosmetics, and medications, and advised to check for alternative names such as “natural red 4,” “E120,” “CI 75470,” and “cochineal extract.”, and to read product labels carefully. Also, patients should be educated on the appropriate use of an epinephrine autoinjector for emergency treatment and be provided with a medical alert card. There is currently no evidence to support desensitization or preventive pharmacotherapy for food additive allergies.

This case report has certain limitations. The absence of an oral food challenge test, considered the gold standard for confirming food allergy, is a notable limitation; however, the patient declined this procedure due to concerns about provoking an anaphylactic reaction. Another limitation of this case report is the absence of manufacturer-provided concentration data for certain commercial preparations, which may affect reproducibility.

## 4 Conclusion

Idiopathic anaphylaxis is a serious condition that necessitates thorough re-evaluation of clinical history and, when indicated, additional diagnostic work-up. Hidden allergens, including additives and colorings commonly present in foods, drugs, and cosmetics, should be considered alongside other possible differential diagnoses. Skin testing with natural substances, such as carmine, may provide useful clues, with confirmation supported by clinical improvement after elimination or by a carefully designed provocation test when risk allows.

## Data Availability

The raw data supporting the conclusions of this article will be made available by the authors, without undue reservation.
